# Laparoscopic Transection of the Pancreatic Neck With Enucleation of Intraductal Papillary Mucinous Neoplasm (IPMN) in the Uncinate Process and Pancreatic Reconstruction

**DOI:** 10.1245/s10434-025-18001-5

**Published:** 2025-08-09

**Authors:** Hengli Gong, Yunqiang Cai, Yanyan Lin, Wenbo Meng

**Affiliations:** 1 The First Clinical Medical College of Lanzhou University, Lanzhou, China; 2 West China Hospital of Sichuan University, Chongqing, China; 3 The First Hospital of Lanzhou University, Lanzhou, China

**Keywords:** Intraductal papillary mucinous neoplasm (IPMN), Enucleation, Pancreatic neck, Transection, Laparoscopy, Pancreatic duct stent, Pancreatic reconstruction, Parenchyma-sparing

## Abstract

**Background:**

Laparoscopic transection of the pancreatic neck with enucleation of intraductal papillary mucinous neoplasm (IPMN) in the uncinate process and pancreatic reconstruction represents a novel surgical approach for managing pancreatic intraductal papillary mucinous neoplasms. This technique may provide a new therapeutic option for patients with benign or low-grade malignant tumors in the pancreatic uncinate process. This study aimed to assess this procedure's short- and long-term clinical outcomes and delineate its technical operative details.

**Methods:**

For a 42-year-old man, branch-duct intraductal papillary mucinous neoplasm (BD-IPMN) was diagnosed. Abdominal contrast-enhanced computed tomography (CT) showed an irregular cystic lesion in the cephalad portion of the pancreatic uncinate process, with no enhancement on post-contrast imaging. Abdominal MRI demonstrated the irregular cystic lesion (38 × 18 mm), showing suspected communication with the pancreatic duct. Endoscopic ultrasound (EUS) showed a multicystic lesion in the pancreatic uncinate process measuring up to 40 mm in maximum diameter, with heterogeneous wall thickness and well-defined margins. The patient subsequently underwent laparoscopic transection of the pancreatic neck with enucleation of IPMN in the uncinate process and pancreatic reconstruction.

**Results:**

The patient experienced a biochemical leak postoperatively, with a daily drainage volume of approximately 20 mL. The drainage volume gradually decreased, and the drain was removed on postoperative day 10, followed by hospital discharge. At this writing, follow-up evaluations have shown no complications such as steatorrhea, secondary diabetes, or dyspepsia. A contrast-enhanced CT scan of the entire abdomen showed no abnormalities. Morphologic and immunohistochemical analyses confirmed the diagnosis of IPMN with mild epithelial dysplasia.

**Conclusion:**

Laparoscopic transection of the pancreatic neck with enucleation of IPMN in the uncinate process and pancreatic reconstruction represents a safe and effective surgical approach for managing pancreatic uncinate process IPMN.

**Supplementary Information:**

The online version contains supplementary material available at 10.1245/s10434-025-18001-5.

Intraductal papillary mucinous neoplasm (IPMN) is a common cystic tumor of the pancreas. According to the internationally recognized evidence-based Kyoto guidelines for IPMN management,^1^ surgical intervention is preferred over long-term surveillance when worrisome features are present, particularly in patients with acute pancreatitis episodes, elevated serum cancer antigen 19-9 (CA19-9) levels, new-onset or acutely exacerbated diabetes within the past year, cyst larger than 30 mm, enhancing mural nodules smaller than 5 mm, thickened cyst walls, main pancreatic duct (MPD) dilation 5 to 10 mm in size, abrupt MPD caliber changes with distal pancreatic atrophy, peripancreatic lymphadenopathy, or a cyst growth rate of 2.5 mm or more per year. Rational surgical management remains indispensable for IPMN treatment.^2^

Although patients with IPMN lacking high-risk stigmata or worrisome features exhibit low malignant progression rates, the long-term risk of pancreatic cancer increases over time, particularly when concurrent pancreatic ductal adenocarcinoma (PDAC) is present, which may be challenging to differentiate clinically.^3^ With the widespread adoption of advanced imaging techniques such as MRI, the detection rate for asymptomatic pancreatic cysts has risen significantly. As a typical benign pancreatic cystic lesion and well-established precursor to pancreatic cancer, IPMN demonstrates an 8 % rate of coexistence with resected PDAC.^4,5^

Located in the pancreatic uncinate process, IPMN poses unique surgical challenges due to its anatomic obscurity. Although pancreaticoduodenectomy (PD) remains the standard procedure for tumors in the pancreatic head or uncinate process,^6–8^ it carries significant risks of postoperative diabetes, exocrine insufficiency, and hepatic steatosis when applied to benign disease.^9^ Recent studies^10^ demonstrate that parenchyma-sparing local enucleation for pancreatic neoplasms achieves lower rates of pancreatic fistula (21.0 %), postoperative hemorrhage (9.9 %), and delayed gastric emptying (4.9 %), with endocrine/exocrine insufficiency rates of 7.1 % and 10.0 %, respectively, significantly lower than for conventional pancreatic resection. Notably, this approach showed 0 % in-hospital mortality and long-term quality of life comparable with that for the general population.

Given the substantial morbidity associated with PD for benign disease, we implemented a novel laparoscopic approach involving pancreatic neck transection, uncinate process IPMN enucleation, and pancreatic reconstruction. Preoperative endoscopic retrograde cholangiopancreatography (ERCP) facilitated a 5-Fr pancreatic duct stent placement. Intraoperatively, laparoscopic transection of the pancreatic neck enabled optimal exposure and complete enucleation of the uncinate process lesion, followed by meticulous main pancreatic duct reconstruction and parenchymal restoration. Both the intraoperative frozen section and final pathology confirmed the diagnosis. Classified as a biochemical leak according to International Study Group for Pancreatic Surgery (ISGPS) criteria,^11^ the postoperative course was managed successfully using drainage, with no complications such as hemorrhage, intra-abdominal infection, pancreatitis, delayed gastric emptying, or endocrine/exocrine dysfunction. This case validated the feasibility of this parenchyma-sparing strategy for benign or low-grade malignant uncinate process lesions.

## Patients and Methods

The authors assume full responsibility for all aspects of this study and confirm adherence to recognized ethical standards. Written informed consent was obtained from the patient.

A 42-year-old man was incidentally found to have a cystic lesion in the uncinate process of the pancreas during a routine physical examination. The asymptomatic lesion demonstrated interval growth on surveillance imaging, prompting surgical intervention. Abdominal contrast-enhanced computed tomography (CT) showed an irregular cystic lesion in the cephalad portion of the pancreatic uncinate process, with no enhancement on post-contrast imaging. Abdominal MRI with magnetic resonance cholangiopancreatography (MRCP) and diffusion-weighted imaging (DWI) demonstrated the irregular cystic lesion (38 × 18 mm), showing suspected communication with the pancreatic duct. Endoscopic ultrasound (EUS) showed a multicystic lesion in the pancreatic uncinate process measuring up to 40 mm in maximum diameter, with heterogeneous wall thickness and well-defined margins. However, endoscopic ultrasound-guided fine-needle aspiration was not performed.

After preoperative ERCP with the placement of a main pancreatic duct stent, the patient underwent laparoscopic pancreatic neck transection with uncinate process IPMN enucleation and pancreatic reconstruction under general anesthesia on 17 August 2024. The pneumoperitoneum was established using a five-port configuration. Systematic exploration of the abdominal and pelvic cavities showed no abnormalities. The gastrocolic ligament was divided for entrance to the omental bursa.

After exposure of the pancreatic body, meticulous dissection along the inferior pancreatic border identified the superior mesenteric vein (SMV). A retro-pancreatic tunnel was created posterior to the pancreatic neck. The pancreatic neck was divided anterior to the SMV using ultrasonic shears, with scissors transection of the main pancreatic duct. The pre-placed ductal stent was preserved. The pancreatic head was mobilized to expose the uncinate process. Ultrasonic shears dissection and sharp scissor techniques achieved complete enucleation of the multilocular cystic tumor. Intraoperative exploration confirmed communication with the main pancreatic duct. The defect created in the main pancreatic duct after transection of the multilocular cystic tumor at its junction was primarily repaired with interrupted 4-0 polydioxanone sutures (PDS).

Frozen-section analysis confirmed IPMN with low-grade intraepithelial neoplasia and negative margins. The teres hepatis ligament was mobilized to reinforce the posterior anastomotic wall, followed by pancreatic reconstruction involving closure of the posterior stump with 4-0 PDS continuous sutures, meticulous anastomosis of the main pancreatic duct using 5-0 PDS interrupted sutures combined with stent placement, and finalization of the anterior stump closure with 4-0 PDS continuous sutures to ensure structural integrity. After confirmation of hemostasis, three drainage catheters were placed near the anastomosis and right hepatorenal space.

## Results

The operative duration was 180 min, with an estimated blood loss of 40 mL. The patient experienced a biochemical leak (grade A according to ISGPS criteria) postoperatively, with a daily drainage output of approximately 20 mL. The drainage volume progressively decreased, allowing drain removal on postoperative day 10, which was followed by uneventful discharge.

During the follow-up period, no complications (i.e., steatorrhea, secondary diabetes mellitus, dyspepsia, or hepatic steatosis) were observed*.* A contrast-enhanced CT of the entire abdomen demonstrated normal postoperative anatomy without evidence of recurrence or fluid collections. Histopathologic examination with immunohistochemical profiling confirmed the diagnosis of IPMN exhibiting mild epithelial dysplasia.

## Discussion

The diagnosis of BD-IPMN was unequivocally established for this patient through preoperative evaluations and intraoperative confirmation. The presence of a tumor larger than 3 cm with worrisome features (including cyst growth rate and heterogeneous wall thickness) provided definitive surgical indications. The patient's persistent anxiety further supported this decision, repeated clinical consultations, and the strong preference for parenchyma-sparing surgical management.

Parenchyma-preserving local enucleation confers long-term quality-of-life benefits by minimizing metabolic complications and reducing psychological burden, thereby enhancing postoperative psychosocial adaptation, a critical consideration for benign or low-grade malignant lesions. In this case, the BD-IPMN demonstrated both multilocular morphology and direct communication with the main pancreatic duct (MPD), features histologically verified during surgery.

To date, limited literature exists on parenchymal-sparing enucleation for BD-IPMN with such anatomic complexity. The successful execution of this procedure provides a novel technical reference for localized tumor enucleation while preserving pancreatic function.

Endoscopic pancreatic duct-stenting represents an effective adjunct in local pancreatic head enucleation for protecting the biliopancreatic ducts, reducing postoperative pancreatic fistula, and preventing long-term ductal stenosis. This technique enhances intraoperative tactile feedback to facilitate MPD localization and promptly identifies intraoperative MPD injuries. Postoperatively, the stent ensures pancreatic fluid drainage, thereby mitigating severe pancreatic leaks. However, procedure-related pancreatitis and intraoperative pancreatic edema remain significant limitations of ERCP-guided stenting.^12^ Consequently, optimizing stent placement timing and executing meticulous, technically refined maneuvers are paramount for achievement of successful outcomes, necessitating further clinical investigation and validation.

Crippa et al.^13^ and Beger et al.^14^ reported that for benign pancreatic lesions such as BD-IPMN, a minimum distance of 2 to 3 mm from the main MPD is essential during local enucleation to significantly reduce the risk of MPD injury and postoperative pancreatic fistula. In a retrospective analysis of 52 patients by Brient et al.,^15^ a tumor-to-MPD distance less than 2 mm was identified as the sole risk factor for pancreatic fistula after local enucleation. Heeger et al.^16^ similarly observed a significant increase in postoperative pancreatic fistula rates when tumors were ≤3 mm from the MPD. Weilin et al.^17^ noted that local enucleation of benign pancreatic tumors carries a higher postoperative pancreatic fistula rate (up to 33 %) than pancreaticoduodenectomy, primarily due to deep tumor locations and proximity to the MPD, leading to ductal injury. However, Strobel et al.^18^ found no significant association between pancreatic fistula occurrence and tumor size or MPD distance in their study of 166 patients undergoing local pancreatic enucleation. Other studies^19^ suggest that although overall pancreatic fistula rates remain higher in benign tumor enucleation, the incidence of clinically relevant (grade B/C) fistulas and severe complications remains low. Critical factors for minimizing postoperative leaks include maintaining adequate tumor-to-MPD distance and intraoperative identification of ductal injuries.

In the current case, we implemented a novel surgical approach involving transection of the pancreatic neck and MPD to expose the tumor. The lesion was located in the cephalad portion of the pancreatic uncinate process, concealed within a complex anatomic region posterior to the SMA and superior mesenteric vein (SMV), anterior to the SMV and portal vein, and adjacent to the inferior vena cava. The proximity to the duodenal, common bile duct, pancreatic ductal, and pancreaticoduodenal arterial arcade structures further increased surgical complexity. Preserving duodenal blood supply and perfusion to the pancreatic head/uncinate process was prioritized to minimize postoperative complications. By splitting the pancreatic neck, we achieved improved exposure of the uncinate mass while reducing intraoperative bleeding and optimizing visualization. After tumor enucleation, meticulous MPD repair and pancreatic reconstruction were performed. This technique minimized vascular ligation between the pancreatic head and duodenum, preventing ischemic complications such as necrosis.

The procedure demands advanced surgical expertise for precise ductal reconstruction and pancreatic restoration. This innovative approach provides a valuable alternative for local enucleation of benign or low-grade malignant tumors in the pancreatic uncinate region.

In summary, laparoscopic transection of the pancreatic neck with enucleation of a benign uncinate process tumor followed by pancreatic reconstruction represents a novel approach for localized enucleation of tumors in this anatomically challenging region. Integrating endoscopic pancreatic duct-stenting may enhance procedural safety. However, this preliminary case study highlights the need to validate long-term benefits through large-scale, multicenter studies. Furthermore, the successful application of this technique demands substantial surgical expertise in complex pancreatic dissection and meticulous ductal reconstruction.

## Supplementary Information

Below is the link to the electronic supplementary material.

Supplementary file 1 (MP4 250964 KB)
